# The effect of antenatal care on use of institutional delivery service and postnatal care in Ethiopia: a systematic review and meta-analysis

**DOI:** 10.1186/s12913-018-3370-9

**Published:** 2018-07-24

**Authors:** Gedefaw Abeje Fekadu, Getachew Mullu Kassa, Abadi Kidanemariam Berhe, Achenef Asmamaw Muche, Nuradin Abusha Katiso

**Affiliations:** 1 School of Public Health, College of Medicine and Health Sciences, Bahir Dar University, P.O.Box 79, Bahir Dar, Ethiopia; 2 College of Health Sciences, Debre Markos University, Debre Markos, Ethiopia; 3 College of Medicine and Health Science, Adigrat University, Adigrat, Tigray Ethiopia; 4 Department of Epidemiology and Biostatistics, Institute of public health, University of Gondar, Gondar, Ethiopia; 5 Department of Nursing, College of Health Sciences and Medicine, Woliata Sodo University, Woliata Sodo, Ethiopia

**Keywords:** Antenatal care, Postnatal care, Institutional delivery, Ethiopia, Meta-analysis

## Abstract

**Background:**

Although there are many initiatives to improve maternal health services use, utilization of health facility delivery and postnatal care services is low in Ethiopia. Current evidence at global level showed that antenatal care increases delivery and postnatal care services use. But previous studies in Ethiopia indicate contrasting results. Therefore, this meta-analysis was done to identify the effect of antenatal care on institutional delivery and postnatal care services use in Ethiopia.

**Methods:**

Studies were searched from databases using keywords like place of birth, institutional delivery, and delivery by a skilled attendant, health facility delivery, delivery care, antenatal care, prenatal care and postnatal care and Ethiopia as search terms. The Joanna Briggs Critical Appraisal Tools and the Preferred Reporting Items for Systematic Review and Meta-Analyses were used for quality assessment and data extraction. Data analysis was done using STATA 14. Heterogeneity and publication bias were assessed using *I*^*2*^ test statistic and Egger’s test of significance. Forest plots were used to present the odds ratio (OR) with 95% confidence interval (CI).

**Result:**

A total of 40 articles with a total sample size of 26,350 were included for this review and meta-analysis. Mothers who had attended one or more antenatal care visits were more likely (OR = 4.07: 95% CI 2.75, 6.02) to deliver at health institutions compared to mothers who did not attend antenatal care. Similarly, mothers who reported antenatal care use were about four times more likely to attend postnatal care service (OR 4.11, 95% CI: 3.32, 5.09).

**Conclusion:**

Women who attended antenatal care are more likely to deliver in health institutions and attend postnatal care. Therefore, the Ethiopian government and other stakeholders should design interventions that can increase antenatal care uptake since it has a multiplicative effect on health facility delivery and postnatal care services use. Further qualitative research is recommended to identify why the huge gap exists between antenatal care and institutional delivery and postnatal care services use in Ethiopia.

## Background

About 303,000 mothers died from pregnancy and childbirth related causes in 2015. Majority (99%) of the deaths occurred in developing countries. Most of these deaths were from Sub-Sahara Africa [1, 2]. In Ethiopia, an estimated 11,000 mothers died due to pregnancy and childbirth related causes in 2015 [1, 3].

Globally, the major causes for maternal mortality are obstetric hemorrhage, hypertension, abortion, sepsis, HIV, preexisting medical disorders and other indirect causes like anemia [2, 4–6]. These are also causes of death for Ethiopian mothers [3, 7–10]. Most causes of maternal and child deaths are preventable or treatable with proven, cost-effective interventions [11–17]. A study conducted in India showed that 90% of maternal deaths would have been prevented if immediate decisions and appropriate care had been given at the time of delivery [18]. Provision of effective delivery care can prevent 113,000 maternal deaths annually [19].

Antenatal, delivery and postnatal care are among the key health sector interventions for maternal and child survival [20–28]. Many studies identified that antenatal care interventions reduce maternal and child mortalities and morbidities [29–34]. Institutional delivery can reduce the risk of neonatal mortality by 29% in low and middle-income countries [35, 36]. A study done in Southern and central India showed that increased institutional delivery is associated with decrease in stillbirth and perinatal mortality [37]. Similarly, skilled attendant at delivery can prevent and treat life-threatening conditions that may occur at the time of delivery [38–41]. Postnatal care is also a crucial time to tackle most causes of maternal and child mortality [42–45].

The Ethiopian government developed many strategies and programs to improve maternal and child health. For example, all maternal health services are provided free in Ethiopia [45–48]. The Health Extension program is another strategy which brought a tangible effect on family health [48, 49]. The Ethiopian government set an ambitious plan to increase four or more ANC visits, delivery and postnatal care services use to 95, 90, and 95% respectively at the end of 2020 although the current level of these services use is low [50, 51].

Antenatal care is an opportunity to promote mothers to use other maternal health services [34, 45, 52, 53]. Women who attended ANC are expected to use health facility delivery and attend postnatal care services. Yet, the situation is different in Ethiopia. According to the 2016 Ethiopian demographic and health survey, the proportion of women who attended ANC, health facility delivery and postnatal care is low compared to the national targets. Moreover, the proportion of mothers who delivered at health institutions (26%) and attended postnatal care (17%) is much lower than those who attended ANC (64%) [50, 51]. Therefore, this review and meta-analysis were done to identify the effect of ANC on institutional delivery and postnatal care services use in Ethiopia. The result of this review and meta-analysis will help to identify whether antenatal care attendance has an effect on health facility delivery and postnatal care services use in Ethiopia.

## Methods

### Search strategy

We used the EndNote software and searched databases to retrieve studies for this review and meta-analysis. The search terms used were: place of birth, institutional delivery, delivery by a skilled attendant, health facility delivery, delivery care, antenatal care, prenatal care and postnatal care and Ethiopia. The main databases searched were PUBMED, MEDLINE, Google Scholar, web of science and African journal online (AJOL). After identifying the key literatures, their references were screened to retrieve additional articles.

### Evaluation of evidence

To evaluate the quality of the papers, the Joanna Briggs Critical Appraisal Tools for review and meta-analysis was used. The AACODS (Authority, Accuracy, Coverage, Objectivity, Date, and Significance) was used to evaluate the quality of the articles [54, 55].

### Inclusion criteria

The following criteria were used to include studies in this meta-analysis.

- Design: studies with all study design
- Publication status: both published and unpublished reports
- Language: literatures reported or published in English
- Publications or report year: papers published or reported up to September 05, 2017
- Place of study: studies that were conducted in Ethiopia regardless of the study setting (community-based or institution based).
- Outcome reported: studies that reported the study outcomes (ANC and institutional delivery or ANC and postnatal care) or both

### Data abstraction

This review was conducted from July 15 to September 05, 2017. The review followed the Preferred Reporting Items for Systematic Review and Meta-Analyses (PRISMA) flow chart to identify and select relevant studies for this analysis. Initially, duplicated retrievals were removed. Then, studies whose titles were irrelevant for this study were excluded. After that, the abstracts were assessed and screened based on the exposure and outcome variables. At this stage, studies that were not relevant for this analysis were excluded. For the remaining articles, the full text was assessed. The eligibility of these articles was assessed based on the pre-set inclusion criteria. When articles did not have adequate data, corresponding authors of the research articles were contacted. All authors conducted the review independently and an agreement was reached through discussion when needed.

### Heterogeneity and publication bias

Heterogeneity among the included studies was checked by using *I*^*2*^ test statistic [56]. Heterogeneity was declared at *p* ≤ 0.05. Publication bias was also checked by using Egger’s test. A *p*-value of less than 0.05 was used to declare statistical significance of publication bias [57]. For studies which showed the presence of publication bias, the Duval and Tweedie nonparametric trim and fill analysis was conducted to account for the publication bias [58].

### Data analysis

The analysis to identify the effect of ANC visits on institutional delivery service use was divided into two parts. The first analysis was to identify the effect of one or more ANC visits on institutional delivery service use and the second was an analysis of the effect of four or more ANC visits on institutional delivery service use.

Data were extracted from each study using data abstraction format prepared on Microsoft Excel. Then, the data were exported to STATA 14 for meta-analysis.

## Results

### Description of the studies

**Fig. 1:**
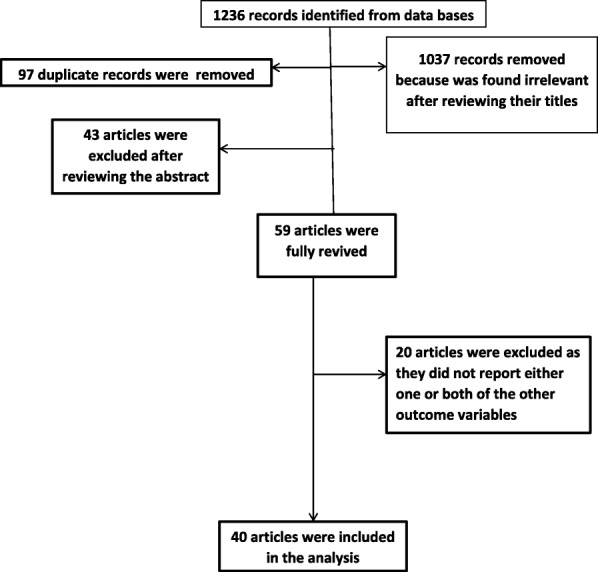
Diagrammatic presentation of the procedure for selection of studies included to study the effect of antenatal care on institutional delivery service use and postnatal care in Ethiopia

A total of 1236 records related to the review topics were identified. Ninety articles were removed because they were duplicates. Another 1139 articles were removed from the list after screening their title and abstracts. Then, full article review and screening was done for 59 studies. From these, a total of 20 articles were excluded for not reporting one or more of the outcome variable. Finally, 40 studies were included in the analysis (Fig. 1, Tables 1, 2 and 3).

**Table 1:** Characteristics of studies included to study the effect of ANC visit on institutional delivery service use in Ethiopia

S.No	Author and Year	Study area	Study period	Study design	Sample size	ANC attendance	Institutional delivery	
							Yes	No
1	Tekelab et al., 2015 [62]	East Wollega, Oromia	January, 2015	CB cross sectional	801	Yes	240	254
						No	77	277
2	Tsegay et al., 2013 [63]	Samri-Saharity District, Tigray	Not reported	CB cross sectional	1115	Yes	39	563
						No	7	504
3	Hailu et al., 2014 [64]	Tsegedie District, Tigray	November 2012 to June 2013	CB cross sectional	485	Yes	124	140
						No	29	192
4	Feyissa et al., 2014 [59]	East Wollega, Oromia	September to October, 2013	Unmatched case control	320	Yes	73	168
						No	7	72
5	Mengesha et al., 2013 [60]	Dabat District, Amhara	October 2009 to August, 2012	Nested case control	1065	Yes	213	852
						No	152	159
6	Abebe et al., 2012 [61]	Bahir Dar Special Zone, Amhara	July, 2010	Unmatched case control	324	Yes	205	57
						No	11	51
7	Abeje et al., 2014 [65]	Bahir Dar city administration,	Jun to July, 2012	CB cross sectional	484	Yes	359	54
						No	20	14
8	Asres et al., 2015 [66]	Sheka zone, SNNP	February to March, 2008	CB cross sectional	554	Yes	319	126
						No	13	96
9	Odo et al., 2014 [67]	Goba town, Oromia	April, 2013	CB cross sectional	580	Yes	247	231
						No	17	67
10	Amano, 2012 [68]	Munisa Woreda, Oromia	April, 2011	CB cross sectional	855	Yes	74	223
						No	31	527
11	Teferra et al., 2012 [69]	Sekela District, Amhara	August, 2010	CB cross sectional	371	Yes	42	3
						No	206	120
12	Worku et al., 2013 [70]	North Gondar Zone, Amhara	January to March, 2012	CB cross sectional	1668	Yes	103	58
						No	145	170
13	Bayu et al., 2015 [71]	Southern Zone, Tigray	January to August 2014	CB follow up	522	Yes	263	82
						No	68	52
14	Melaku et al., 2014 [72]	Kilite Awulalo, Tigray	September 2009 to August 2012	CB, longitudinal	2361	Yes	536	1270
						No	106	449
15	Abera et al., 2011 [73]	Arsi Zone, Oromia	February to March, 2006	CB cross sectional	1089	Yes	162	482
						No	14	416
16	Tura G, 2008 [74]	Metekel Zone, B/Gumuz	January to March, 2007	CB cross sectional	1060	Yes	108	409
						No	17	504
17	Nigussie et al., 2004 [75]	North Gondar Zone, Amhara	November to December, 2002	CB cross sectional	1248	Yes	147	421
						No	21	653
18	Tura et al., 2014 [76]	Jimma Zone, Oromia	September 2012–April 2013	CB follow up study	3472	Yes	954	1680
						No	110	728
19	Arba et al., 2016 [77]	Wolayta & Dawuro Zones, SNNPR	February to March, 2015	CB cross sectional	1000	Yes	326	435
						No	33	163
20	Bayu et al., 2015 [78]	Debremarkos town, Amhara	January to July, 2012	CB, follow up	422	Yes	232	116
						No	13	31
21	Darega et al., 2016 [79]	Abuna Gindeberet District, Oromia	March, 2013	CB cross sectional	703	Yes	98	481
						No	3	121
22	Demilew et al., 2016 [80]	Dangila district, Amhara	February, 2015	CB cross sectional	780	Yes	134	377
						No	6	246
23	Fikre and Demissie, 2012 [81]	Dodota District, Oromia	January, 2011	CB cross sectional	506	Yes	75	340
						No	17	74
24	Habte and Demissie, 2015 [82]	Cheha District, SNNPR	December 2012 to January 2013	CB cross sectional	845	Yes	251	483
						No	2	80
25	Kebede et al., 2013 [83]	Chilga, Amhara	March to June 2012	CB cross sectional	475	Yes	54	218
						No	19	184
26	Kenea and Jisha, 2017 [84]	Dale Wabera District, Oromia	2014	CB cross sectional	588	Yes	215	185
						No	45	122
27	Kidanu et al., 2017 [85]	Dembecha District, Amhara	March, 2015	CB cross sectional	700	Yes	6	45
						No	223	400
28	Tadele & Lamaro, 2017 [86]	Bench Maji, SNNPRS	September, 2015	CB cross sectional	800	Yes	574	109
						No	25	57
29	Wako & Kassa, 2017 [87]	Liben District, Oromia	June, 2015	CB cross sectional	876	Yes	76	444
						No	34	237
30	Yigezu and Kitila, 2015 [88]	Jimma town, Oromia	February to April, 2014	CB cross sectional	281	Yes	165	63
						No	18	31

*CB* Community based

**Table 2:** Characteristics of studies included to study the effect of number of ANC visits on institutional delivery service use in Ethiopia

S. No	Author and year	Study area	Study period	Study design	Sample size	Number of ANC visits	Institutional delivery	
							Yes	No
1	Hailu et al., 2014 [64]	Tsegedie District, Tigray	November 2012 to June 2013	CB cross sectional	485	≥4^+^	29	14
						< 4	102	126
2	Feyissa et al., 2013 [59]	East Wollega, Oromia	September to October 2013	Unmatched case control	320	≥4^+^	48	79
						< 4	25	89
3	Mengesha et al., 2013 [60]	Dabat District, Amhara	October 2009 to August 2012	Nested case control	1065	≥4^+^	152	159
						< 4	61	693
4	Odo et al., 2014 [67]	Goba town, Oromia	April, 2013	CB cross sectional	580	≥4^+^	50	32
						< 4	196	200
5	Tura et al., 2014 [76]	Jimma zone, Oromia	September 2012 to April 2013	CB follow up study	3472	≥4^+^	633	595
						< 4	321	1085
6	Alemayehu & Mekonnen, 2015 [89]	Ankasha Gagusa woreda, Amhara	February, 2014	CB cross sectional	373	≥4^+^	23	22
						< 4	41	199
7	Kasaye et al., 2017 [90]	Debremarkos town, Amhara	January, 2016	CB cross sectional	518	≥4^+^	154	14
						< 4	221	113
8	Tadele and Lamaro, 2017 [86]	Bench Maji, SNNPRS	September, 2015	CB cross sectional	800	≥4^+^	427	21
						< 4	147	88
9	Desalegn et al., 2014 [91]	Fogera District, Amhara	February – April, 2013	CB cross sectional	412	≥4^+^	61	42
						< 4	65	231
10	Kibret, 2015 [92]	Gozamen District, Amhara	March to April, 2014	CB cross sectional	499	≥4^+^	44	48
						< 4	79	326

*CB* Community based

**Table 3:** Characteristics of studies included to study the effect of antenatal care on post natal follow up in Ethiopia

S.No	Author and year	Study area	Study period	Study design and setting	Sample size	ANC attendance	PNC attendance	
							Yes	No
1	Tesfahun et al., 2014 [93]	Gondar Zuria district, Amhara	April–August 2011	Community based cross sectional	836	Yes	550	155
						No	59	56
2	Darega et al., 2016 [79]	Abuna Gindeberet District, Oromia	March, 2013	Community based cross sectional	703	Yes	210	369
						No	13	111
3	Limenih et al., 2016 [94]	Debremarkos town, Amhara	November, 2014	Community based cross sectional	588	Yes	138	163
						No	59	228
4	Birhanu et al., 2016 [95]	Addis Ababa	April–May, 2016	Institution based cross sectional	422	Yes	273	139
						No	4	6
5	Hordofa et al., 2015 [96]	Dembecha District, Amhara	July–August, 2013	Community based cross sectional	788	Yes	234	333
						No	22	147
6	Abosse et al. 2015, [97]	Hadya Zone, SNNPRS	January–February 2009	Community based cross sectional	710	Yes	154	442
						No	3	92

The studies were conducted from 2011 to 2017. Most of the studies were from the four major regions of Ethiopia, 11 from Oromia, 17 from Amhara, 4 from Tigray and 5 from South Nations, nationalities and people’s regional state. The sample size of the included studies ranged from 281 to 3472 participants. In terms of study design, all except three were cross-sectional (Tables 1, 2 and 3).

### Effect of ANC on institutional delivery service use

A total of 30 studies with 26,350 sample size were included to estimate the effect of ANC on institutional delivery service use. The study populations for all the 30 studies were reproductive-age women who were pregnant or had given birth within 5 years of the survey. The studies were conducted from 2004 to 2016 (Table 1). Three of the studies were case-control [59–61] and the remaining 27 studies were community-based cross-sectional or follow up studies [62–88].

**Fig. 2:**
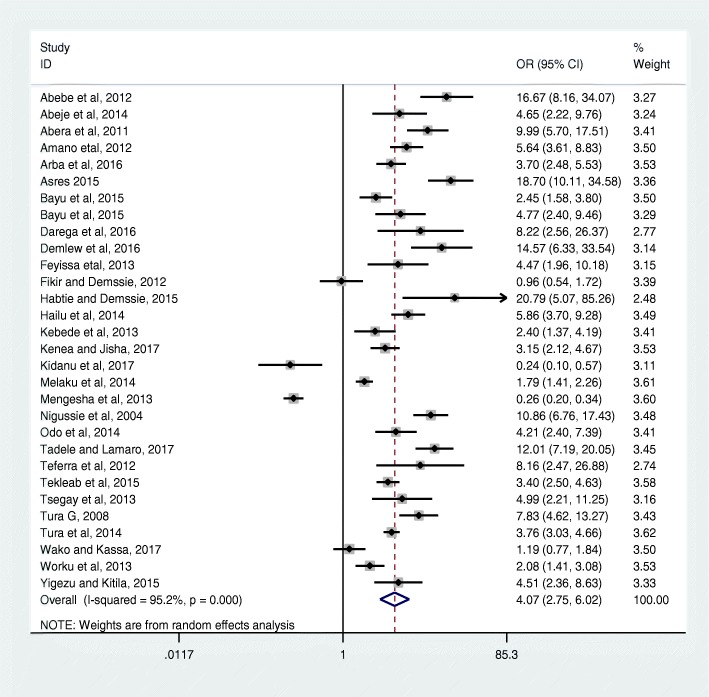
Effect of any antenatal care visit on institutional delivery service use in Ethiopia

This analysis identified that mothers who had one or more antenatal care visits were about four times more likely (OR = 4.07: 95% CI 2.75, 6.02) to deliver at health facilities compared to mothers who had not attended ANC (Fig. 2).

**Fig. 3:**
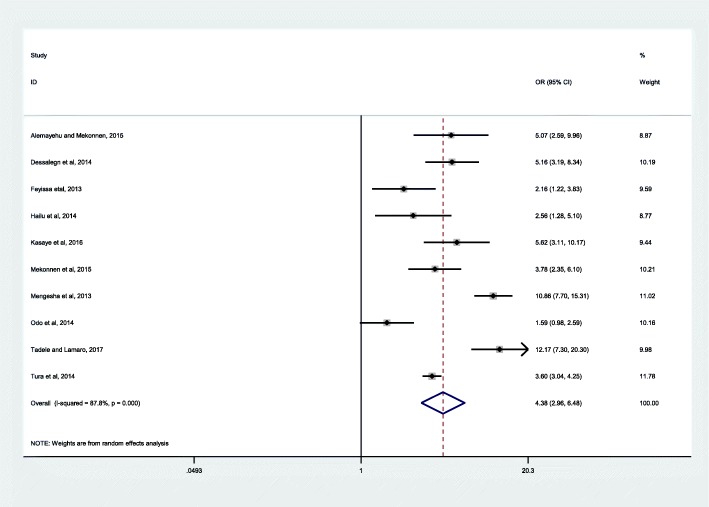
Effect of four or more antenatal care visits on institutional delivery service use in Ethiopia

Additionally, ten articles were included to assess the effect of four or more ANC visits on institutional delivery service use. The studies were conducted from 2013 to 2017. The total number of women included in this analysis was 8524. Two of the studies were case-control [59, 60] and the other eight were cross-sectional studies [64, 67, 76, 86, 89–92]. The sample size of the studies ranged from 320 to 3472. The studies included in this subgroup analysis showed high heterogeneity (*I*^*2*^ = 87.8, *P* ≤ 0.001) but non-significant publication bias (Egger’s test = 0.780). Using the random effect model analysis, women who had four or more ANC visits were 4.38 times more likely to deliver in health facilities compared to women who reported fewer ANC visits (OR 4.38, 95% CI: 2.96, 6. 48) (Fig. 3).

### Effect of ANC on postnatal care service use

**Fig. 4:**
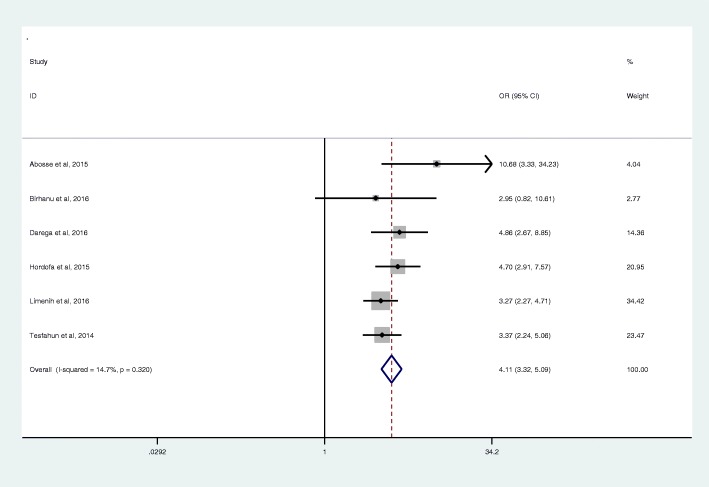
Effect of antenatal care visit on postnatal care service use in Ethiopia

Six articles with a total sample size of 4047 women were included in this analysis. All except one (institution based) were community-based cross-sectional studies [79, 93–97]. There was no statistically significant heterogeneity and publication bias among the studies (*I*^*2*^ = 14.7, *P* = 0.320 and Egger’s test = 0.231, respectively). The analysis indicated that mothers who attended ANC were about four times more likely to use postnatal care service (OR 4.11, 95% CI: 3.32, 5.09) (Fig. 4).

## Discussion

Antenatal care has been used as a strategy to reduce maternal and neonatal morbidities and mortalities. Various approaches and strategies have been implemented to improve the effectiveness of ANC in developing countries [52, 98, 99]. Currently, most developing countries including Ethiopia are using the focused ANC approach which was developed by WHO [100, 101].

This study identified that women who attended ANC were about four times more likely to use institutional delivery services. This finding was in line with a meta-analyses conducted in Africa [102, 103] and DHS based data analysis in Nigeria [104]. The reason for this finding is that ANC is an opportunity for health promotion [105]. Therefore, women who attended ANC are more likely to have better information about benefits of institutional delivery service use and this may have impacted the subsequent health service use. Additionally, pregnant women attending ANC have the chance to acclimatize to the health facility environment. This may have helped them avoid unnecessary fear and stress related to institutional delivery service use. Furthermore, mothers who attended antenatal care are more likely to be better informed about danger signs and obstetric complications which may arise during labor and delivery. Antenatal care is also an opportunity for a pregnant woman to establish an informal forum which will help them to discuss and share information about their pregnancies and benefits of health facility delivery [46, 50, 57, 58].

The subgroup analysis showed four or more ANC visits had a similar effect on health facility delivery compared to fewer ANC visits. The reason for this may be that health professionals in developing countries provide all the information and health promotion activities needed for the mother on the first visit to avoid missed opportunities as the woman’s return for the subsequent visits is not guaranteed [51, 105].

The current review also found that women who attended antenatal care were more likely to use postnatal care services. This finding is similar to studies conducted in Nigeria, Nepal, and Zambia [104, 106, 107]. It is theoretically plausible to think that mothers who attended ANC had received adequate counseling and information about postnatal care during the ANC session. Additionally, women may set birth plans in consultation with the ANC provider which in turn will increase delivery and postnatal service use [108].

This review had large sample size, which meant that it could detect the effect of ANC on institutional delivery and postnatal care services use. The analysis included all studies conducted in Ethiopia. But this meta-analysis does not address other factors that affect institutional delivery service use and postnatal care. In addition, this meta-analysis did not answer why institutional delivery and PNC services use remained low compared to ANC services use in Ethiopia. Evidence to identify the effect of ANC on PNC is limited. Therefore, we recommended further studies to identify the root cause for the huge difference in the proportion of women who attended ANC and PNC.

## Conclusion

This review and meta-analysis revealed that mothers who attended ANC are more likely to use institutional delivery service and postnatal care. Mothers who attended ANC visits were more likely to deliver at health institutions. Similarly, women who attended ANC were more likely to attend postnatal care services. Therefore, the Ethiopian government and other stake holders need to exert collaborative effort to increase ANC service use since it has multiplicative on delivery and postnatal care services use.

## Abbreviations

AIDS: Acquired immune deficiency syndrome
ANC: Antenatal care
DHS: Demographic and health survey
EDHS: Ethiopian demographic and health survey
HIV: Human immune virus
OR: Odds ratio
SSA: Sub-Saharan Africa
UN: United Nations
UNICEF: United Nations International Children’s Emergency fund
WHO: World Health Organization
